# Acute Dysentery

**Published:** 1899-09

**Authors:** 


### Abstract.

### ACUTE DYSENTERY.

Dr. St. J. V. Graham (*Georgia Journal of Medicine and Surgery*, July, 1899,) states that the drug treatment of acute dysentery resolves itself into five or six drugs—calomel, opium, ipecac, tannopin, salines and quinine. If the case is seen early when diarrhea is present, with a lead-colored or brown tongue, much benefit may be derived from giving calomel, a quarter-grain every fifteen minutes, until six, eight or ten doses are taken. An acid saline is then administered, after which bile usually begins to flow. This is nature's antiseptic, and no chemical compound or so-called intestinal antiseptic can be compared with it. After this has been kept up for a sufficient time for the exigencies of the case, tannopin should be administered, combined with ipecac and opium, in the form of Dover's powder, or of each drug in simple powder combination. Tannopin should be given in ten or fifteen grain doses every two and one-half or three hours. An ice bag over the belly is preferred by the writer to any form of poultice. If necessary the bowels are irrigated with a bisulphate of quinine solution—one teaspoonful to a quart of cold water. Very little quinine will be absorbed, for it will not stay in long enough. The diet should be carefully adjusted to suit individual peculiarities and the stomach digestion. Stimulants should be used as indicated. The above treatment, which is indicated in acute cases, has proved very successful. In chronic cases, however, an essentially different drug treatment should be resorted to.

